# SARS‐CoV‐2 re‐infection versus prolonged shedding: A case series

**DOI:** 10.1111/irv.12879

**Published:** 2021-06-04

**Authors:** Erin G. Nicholson, Vasanthi Avadhanula, Sonia Fragoso, Rachel Stroh, Xunyan Ye, Nanette Bond, Patricia Santarcangelo, John Stroh, Pedro A Piedra

**Affiliations:** ^1^ Department of Molecular Virology and Microbiology Baylor College of Medicine Houston TX USA; ^2^ Department of Pediatrics Baylor College of Medicine Houston TX USA; ^3^ School of Medicine Baylor College of Medicine Houston TX USA; ^4^ Department of Emergency Medicine St. Luke's Health Houston TX USA

**Keywords:** re‐infection, SARS‐CoV‐2, shedding

## Abstract

Since the start of the SARS‐CoV‐2 pandemic, it has been difficult to differentiate between SARS‐CoV‐2 re‐infection and prolonged RNA shedding. In this report, we identified patients with positive RT‐PCR results for SARS‐CoV‐2 ≥70 days apart. Clinical and laboratory data were collected and criteria were applied to discern whether the presentation was consistent with SARS‐CoV‐2 re‐infection or prolonged viral RNA shedding. Eleven individuals met the initial testing criteria, of which, seven met at least one criteria for re‐infection and four were consistent with prolonged RNA shedding. These data demonstrate the need for criteria to differentiate SARS‐CoV‐2 re‐infection from prolonged RNA shedding.

## 1 INTRODUCTION

Since the beginning of the severe acute respiratory syndrome coronavirus‐2 (SARS‐CoV‐2) pandemic, the potential for re‐infection with the SARS‐CoV‐2 virus has been a concern. Endemic coronaviruses are known to re‐infect individuals in the same year, so it is plausible that natural infection with SARS‐CoV‐2 would similarly provide short‐lived immunity. However, due to prolonged viral RNA shedding of the SARS‐CoV‐2 virus, it has been difficult to differentiate prolonged shedding from true re‐infection.^1^ This has changed in recent months when several reports emerged of individuals re‐infected with genetically different lineages of SARS‐CoV‐2.^2^, ^3^ These reports represent confirmation that, while uncommon, re‐infection in the span of several months with SARS‐CoV‐2 can occur.

Currently, the Center for Disease Control (CDC) does not recommend testing individuals for re‐infection less than 90 days since symptom onset.^4^, ^5^ However, detailed SARS‐CoV‐2 real‐time reverse transcriptase polymerase chain reaction (RT‐PCR) kinetics data appear to describe a cessation of viral RNA shedding after 70 days in the majority of individuals (though exceptions have been described).^6^, ^7^, ^8^ In this brief report, we describe the clinical characteristics and laboratory results of individuals with multiple positive SARS‐CoV‐2 RT‐PCR results that extend beyond 70 days.

## 2 METHODS

The data described were obtained from two sources. The first is from our diagnostic operation for SARS‐CoV‐2 testing in a Clinical Laboratory Improvement Amendments (CLIA) Certified Respiratory Virus Diagnostic Laboratory (ID#: 45D0919666) that began on March 18, 2020.^9^ The second source came from RT‐PCR positive SARS‐CoV‐2 patients who enrolled in our ongoing study to evaluate antibody response kinetics after their primary infection. We included patients with 2 or more positive RT‐PCR tests for SARS‐CoV‐2 that were at least 70 days apart in order to fully capture a spectrum of patients with prolonged viral RNA shedding vs re‐infection.

Based on proposed re‐infection criteria in the literature, we defined three separate criteria to classify a patient as having a re‐infection with SARS‐CoV‐2. The criteria are as follows: (1) a positive SARS‐CoV‐2 RT‐PCR test at least 90 days after individuals first became symptomatic with SARS‐CoV‐2 and subsequently become asymptomatic,^4^ (2) at least 2 negative RT‐PCR tests for SARS‐CoV‐2 between two positive tests,^10^, ^11^ and (3) at least one negative RT‐PCR test for SARS‐CoV‐2 between two positive tests more than 28 days apart.^12^ For our analysis, only one criterion was needed to be considered a probable re‐infection.

We collected demographic, laboratory, and clinical data from retrospective chart review and performed phone interviews using a structured questionnaire. The protocol was approved by the institutional review board at Baylor College of Medicine.

### 2.1 RT‐PCR assays

Mid‐turbinate swabs were tested for SARS‐CoV‐2 using CDC's EUA for CDC 2019‐Novel Coronavirus (2019‐nCoV) RT‐PCR Diagnostic Panel (Primers and probes target the nucleocapsid (N) gene (N1 and N2) of SARS‐CoV‐2 and ribonuclease P (RNase‐P) gene). The samples were considered positive if N1, N2, and RNaseP cycle threshold (CT) values were <40.^9^

### 2.2 IgG assay

The humoral IgG anti‐spike enzyme‐linked immunosorbent assays (ELISA) were performed as part of our IRB‐approved research study. The SARS‐CoV‐2 full spike (S) protein used in the ELISA was kindly provided by Gale Smith (Novavax).^13^ The samples were considered serologically positive if the optical density (OD) values were ≥0.5 at a serum dilution of ≥1:1024.

## 3 RESULTS

### 3.1 Case series

Our laboratory tested 17,784 samples (from 13,257 patients) for SARS‐CoV‐2 via RT‐PCR from March 18, 2020 to October 31, 2020. Of those, 1,566 samples (from 981 patients) were positive. Within this cohort, we found 11 patients that had at least two positive SARS‐CoV‐2 RT‐PCR tests that spanned >70 days.

### 3.2 Clinical presentation

Of these 11 individuals, 6 were female with an average age of 49 years (Table 1). The majority of individuals (8 of 11) had at least 1 co‐morbid condition. All patients were symptomatic prior to and at their first positive RT‐PCR test. Symptom durations (when available) ranged from 4 to 28 days. Overall, symptoms were mild and none were hospitalized. Only one individual presented to the ED for shortness of breath. The majority of RT‐PCR testing performed after 70 days was performed either as clearance for a procedure or as part of a surveillance program.

**TABLE 1:** Patient demographics and clinical data

Patient number	Age	Gender	Race	Ethnicity	Co‐morbid conditions	0–70 days from first symptoms					>70 days from first symptoms					Re‐infection criteria
						Reason for testing	Symptoms	Level of Care	Duration of symptoms	SARS‐CoV−2 IgG testing (If performed)	Reason for testing	Symptoms	Level of care	Duration of symptoms	SARS‐CoV−2 IgG testing (If performed)	
1	46	M	Asian	Non‐Hispanic	Hypertension, Gastroesophageal Reflux Disease, Plantar Fasciitis	Symptomatic	Fever, Myalgias, Sore Throat, Chills, Headaches, Nausea, Shortness of Breath	Emergency room visit	17 days	1st test: 1:4096 (BCM laboratory)	Surveillance testing	None	Self‐care at home	0 days	2nd test: 1:2048 (BCM laboratory)	1, 2, 3
2	27	F	White	Non‐Hispanic	None	Symptomatic	Congestion, Fatigue, Loss of Taste, Loss of Smell, Headache	^a^	^a^	^a^	Symptomatic	Fever, Chills, Fatigue	^a^	2 days	^a^	1, 2, 3
3	53	M	White	Non‐Hispanic	Hypertension, Sleep Apnea	Symptomatic	Cough, Congestion, Loss of Taste, Loss of Smell	Self‐care at home	15 days	1st test: 1:2048 (BCM laboratory)	Pre‐operative testing	None	Self‐care at home	0 days	2nd test: 1:1024 (BCM laboratory)	1, 3
4	66	F	Black	Non‐Hispanic	Diabetes Mellitus, Rheumatoid Arthritis, Systemic Lupus Erythematosus, Congestive Heart Failure, Renal Disease, Gout, Hypertension	Symptomatic	Fatigue	Self‐care at home	7 days	^a^	Surveillance testing	None	Self‐care at home	0 days	^a^	1
5	73	F	White	Hispanic	Hypertension, Hyperlipidemia, Depression	Symptomatic	Congestion, Sore Throat, Headache	PCP visit	28 days	^a^	Symptomatic	Cough, Shortness of Breath, Congestion, Abdominal Pain, Nausea, Vomiting, Headache	PCP visit	27 days	^a^	1
6	42	F	White	Hispanic	Breast Cancer	Symptomatic	Cough, Shortness of Breath, Fatigue, Loss of Taste, Loss of Smell, Headache, Fever	Self‐care at home	12 days	^a^	Pre‐operative testing	None	Self‐care at home	0 days	1st test: 1:4096 (BCM laboratory)	1
7	36	M	White	Non‐Hispanic	None	Symptomatic	Cough, Fatigue, Nausea, Loss of Smell, Fever	Self‐care at home	4 days	1st test: 1:4096 2nd Test: 1:4096 (BCM laboratory)	Surveillance testing	None	Self‐care at home	0 days	^a^	1
8	48	F	^a^	^a^	Diabetes Mellitus, Polyneuropathy, Seronegative arthritis	Symptomatic	Cough, Fatigue, Myalgias, Sore Throat, Loss of Taste, Loss of Smell	PCP visit	^a^	^a^	Patient desired follow‐up testing	None	Self‐care at home	0 days	1st test: 2.3 (CPL laboratory)	Does not meet criteria
9	64	F	White	Non‐Hispanic	Lupus, Hepatitis C	Symptomatic	Cough, Fatigue, Nausea, Dizziness, Headache, Fever	Self‐care at home	16 days	^a^	Pre‐operative testing	None	Self‐care at home	0 days	^a^	Does not meet criteria
10	47	M	White	Hispanic	Diabetes Mellitus, Polyneuropathy, Depression, Hypertension	Symptomatic	Cough, Congestion, Fatigue, Headache, Fever, Diarrhea, Sore Throat, Loss of Smell, Myalgias, Chills	PCP visit	^a^	^a^	Patient desired follow‐up testing	None	Self‐care at home	0 days	1st test: 2.9 (CPL laboratory)	Does not meet criteria
11	36	M	White	Hispanic	None	Symptomatic	Cough, Shortness of Breath, Fatigue, Diarrhea, Chills, Loss of Taste, Loss of Smell	Self‐care at home	4 days	^a^	Return to work testing	None	Self‐care at home	0 days	^a^	Does not meet criteria

^a^ Information not available.

Only two individuals were symptomatic at the time of their testing >70 days after initial symptoms with their primary SARS‐CoV‐2 infection. Both individuals had complete resolution of symptoms with their primary infection.

### 3.3 Probable re‐infection definition

**FIGURE 1:**
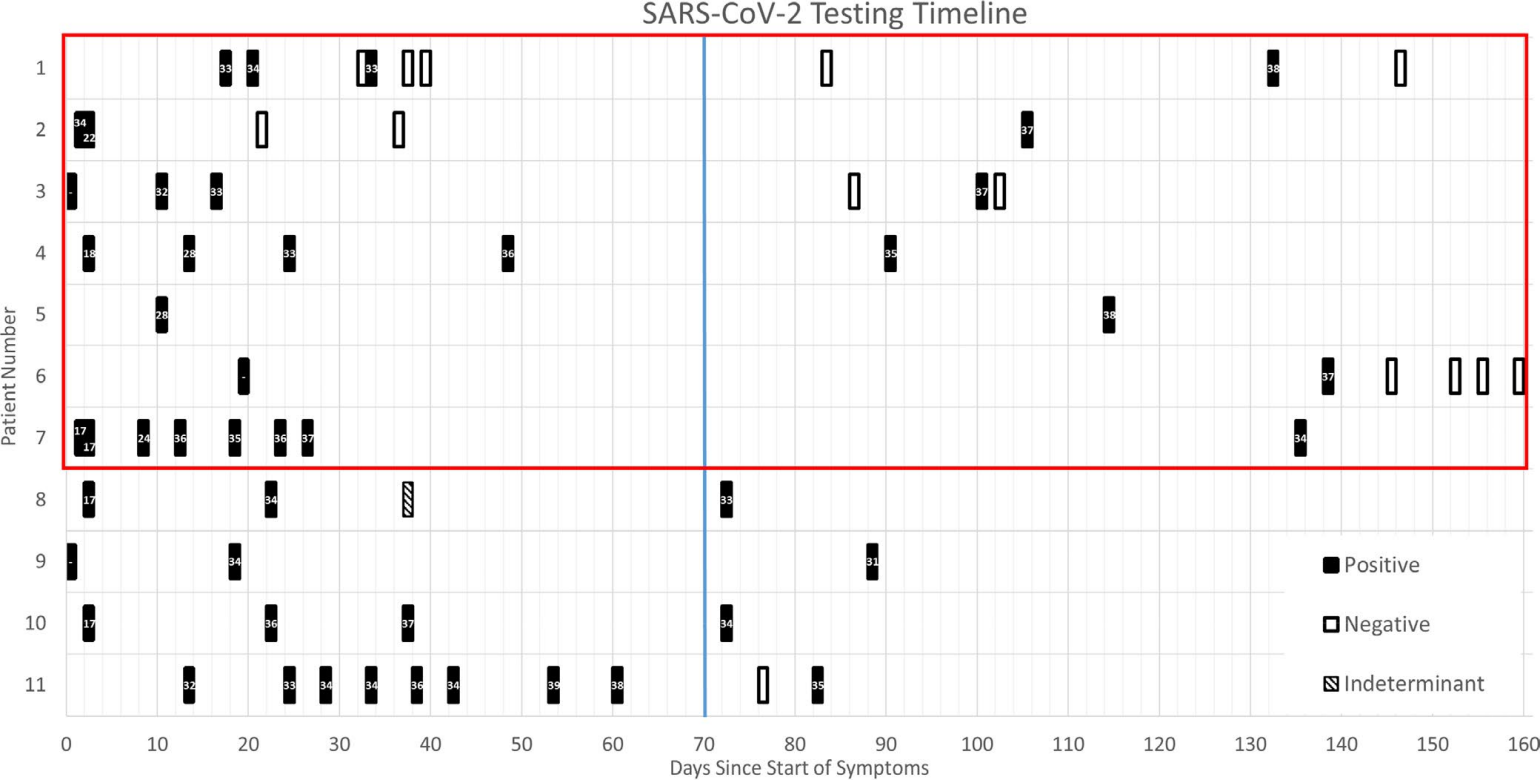
This figure represents the longitudinal SARS‐CoV‐2 RT‐PCR data obtained for our 11 patients included in the cohort. The *x*‐axis defines the days since the start of symptoms for each patient. The *y*‐axis provides the patient number. The black boxes represent a positive RT‐PCR test for SARS‐CoV‐2, the white boxes represent a negative RT‐PCR test for SARS‐CoV‐2, and the hashed boxes represent an indeterminate RT‐PCR test for SARS‐CoV‐2. The cycle threshold values are listed for each positive sample within the black box. The red box represents those with probable SARS‐CoV‐2 re‐infection and the blue line represents the 70‐day cutoff for inclusion in the study

We used three criteria to assess the likelihood of a SARS‐CoV‐2 re‐infection that occurred greater than 70 days after primary infection (described above). We found that 7 of the 11 individuals met at least one criterion for probable re‐infection and all 7 of these individuals met Criteria 1 (see Figure 1 and Table 1). Three (patients 1–3) of the 7 individuals with probable re‐infection had at least one negative test prior to another SARS‐CoV‐2 RT‐PCR positive test, thus fitting Criteria 2 or 3. Viral sequencing was performed, however, none of the samples >70 days out had sufficient genomic material for whole genome sequencing (WGS) or Sanger sequencing (data not shown). Four (patients 8–11) individuals did not meet any of the criteria for re‐infection and were considered to be experiencing prolonged shedding of SARS‐CoV‐2 RNA.

### 3.4 Serology

Six of the 11 individuals had IgG serology testing for SARS‐CoV‐2 (Table 1). Only two individuals (patients 1 and 3) had a paired sample whose sera was collected before and after their probable re‐infection. Neither of these individuals mounted an increase in IgG anti‐S antibody levels after their probable re‐infection (Table 1).

## 4 DISCUSSION

In this case series, we identified 11 individuals who had viral RNA detected from their upper respiratory tract >70 days after their primary SARS‐CoV‐2 infection. We describe two groups: a probable SARS‐CoV‐2 re‐infection group and a probable primary infection group with prolonged viral RNA shedding. Of these 11 individuals, seven were consistent with probable re‐infection, while four did not meet the criteria for probable re‐infection and thus were considered primary infection with prolonged viral RNA shedding. We based this conclusion on several factors. First, two patients had ≥2 negative RT‐PCR tests prior to their probable SARS‐CoV‐2 re‐infection and one patient (patient 3) had one negative test prior to their probable re‐infection. Second, all seven individuals had at least 90 days between their primary SARS‐CoV‐2 infection and their probable SARS‐CoV‐2 re‐infection. We attempted to be more inclusive for re‐infections occurring <90 days after initial symptom onset with primary infection, however, none were identified. Our data support the assertion by the CDC that re‐infection is unlikely to occur within 90 days from onset of symptoms with primary SARS‐CoV‐2 infection.^4^, ^5^

Definitive SARS‐CoV‐2 re‐infection has been confirmed in a small number of cases.^2^, ^3^ Confirmation via WGS demonstrated that the SARS‐CoV‐2 isolate detected with the re‐infection event was sufficiently different from the isolate sequenced from the primary infection. In our population, we were unable to confirm SARS‐CoV‐2 re‐infection cases using WGS or Sanger sequencing of the S gene because the viral RNA quantity isolated during the probable re‐infection events was insufficient despite multiple sequencing attempts. In two cases, we had blood samples collected prior to and after their probable SARS‐CoV‐2 re‐infection. In both cases, we did not observe a fourfold or greater rise in IgG anti‐S antibody levels consistent with an infection. Lack of a significant antibody rise has been reported in another case report with confirmed SARS‐CoV‐2 infection.^2^, ^14^ This lack of antibody response with re‐infection was attributed to the patient's mild illness, which is consistent with our observation that both of our patients were asymptomatic with their re‐infection event.^14^

Early re‐infection or prolonged viral RNA shedding with primary SARS‐CoV‐2 infection might be a manifestation of non‐sterilizing immunity that is generated with the initial infection. Unlike other limited case reports, which have documented recurrence of infections after hospitalization due to SARS‐CoV‐2, this is an ambulatory population with relatively mild symptomatology.^12^ None of our seven patients were hospitalized during their first episode and only two patients were symptomatic with their probable re‐infection event. Patient 5 had the highest severity during her probable re‐infection, and she was also the oldest patient in our cohort. Another study that examined viral kinetics of SARS‐CoV‐2 found that a higher severity of symptoms (ICU admission) significantly decreased a patient's likelihood of prolonged shedding of SARS‐CoV‐2 RNA.^6^ This coupled with observations that individuals with severe cases of SARS‐CoV‐2 infection have increased IgG anti‐S antibody responses compared with mild cases could explain why we see predominantly mild presentations in our cohort resulting in probable re‐infections or primary infection with prolonged viral RNA shedding.^13^, ^15^

### 4.1 Limitations

This was a single site cohort study that reported on approximately seven months of RT‐PCR testing for SARS‐CoV‐2. The low viral RNA content in the samples collected from probable re‐infection cases precluded our ability to confirm the re‐infection event using sequencing.

## 5 CONCLUSIONS

The occurrence of probable re‐infection with SARS‐CoV‐2 in our population seemed to be an uncommon occurrence that was associated with mild symptomatology at the time of their primary infection. Our data does support the CDC's recommendation that testing for SARS‐CoV‐2 re‐infection is not needed prior to 90 days after symptoms arise.

## AUTHOR CONTRIBUTIONS

**Erin G Nicholson:** Conceptualization (equal); Investigation (equal); Methodology (equal); Writing‐original draft (lead); Writing‐review & editing (equal). **Vasanthi Avadhanula:** Conceptualization (equal); Data curation (equal); Investigation (equal); Project administration (equal); Resources (equal); Supervision (equal); Writing‐review & editing (equal). **Sonia Fragoso:** Conceptualization (equal); Data curation (equal); Formal analysis (equal); Investigation (equal); Project administration (equal); Writing‐review & editing (equal). **Rachel Stroh:** Data curation (equal); Investigation (equal); Validation (equal); Writing‐review & editing (equal). **Xunyan Ye:** Conceptualization (equal); Investigation (equal); Methodology (equal); Project administration (equal); Writing‐review & editing (equal). **Nanette Bond:** Data curation (equal); Investigation (equal); Methodology (equal); Project administration (equal); Writing‐review & editing (equal). **Patricia Santarcangelo:** Data curation (equal); Investigation (equal); Methodology (equal); Project administration (equal); Writing‐review & editing (equal). **John Stroh:** Conceptualization (equal); Investigation (equal); Methodology (equal); Writing‐original draft (supporting); Writing‐review & editing (equal). **Pedro A Piedra:** Conceptualization (equal); Investigation (equal); Methodology (equal); Project administration (equal); Resources (equal); Supervision (equal); Writing‐review & editing (lead).

### PEER REVIEW

The peer review history for this article is available at https://publons.com/publon/10.1111/irv.12879.

## Data Availability

The data that support the findings of this study are available on request from the corresponding author. The data are not publicly available due to privacy or ethical restrictions.
